# Electrophysiological Mechanisms Underlying T-Wave Alternans and Their Role in Arrhythmogenesis

**DOI:** 10.3389/fphys.2021.614946

**Published:** 2021-03-04

**Authors:** Tingting You, Cunjin Luo, Kevin Zhang, Henggui Zhang

**Affiliations:** ^1^Key Lab of Medical Electrophysiology, Ministry of Education, Institute of Cardiovascular Research, Southwest Medical University, Luzhou, China; ^2^School of Computer Science and Electronic Engineering, University of Essex, Colchester, United Kingdom; ^3^School of Medicine, Imperial College of London, London, United Kingdom; ^4^Department of Physics and Astronomy, University of Manchester, Manchester, United Kingdom

**Keywords:** T-wave alternans, sudden cardiac death, spatial discordant alternans, arrhythmia, simulation

## Abstract

T-wave alternans (TWA) reflects every-other-beat alterations in the morphology of the electrocardiogram ST segment or T wave in the setting of a constant heart rate, hence, in the absence of heart rate variability. It is believed to be associated with the dispersion of repolarization and has been used as a non-invasive marker for predicting the risk of malignant cardiac arrhythmias and sudden cardiac death as numerous studies have shown. This review aims to provide up-to-date review on both experimental and simulation studies in elucidating possible mechanisms underlying the genesis of TWA at the cellular level, as well as the genesis of spatially concordant/discordant alternans at the tissue level, and their transition to cardiac arrhythmia. Recent progress and future perspectives in antiarrhythmic therapies associated with TWA are also discussed.

## Introduction

**FIGURE 1:**
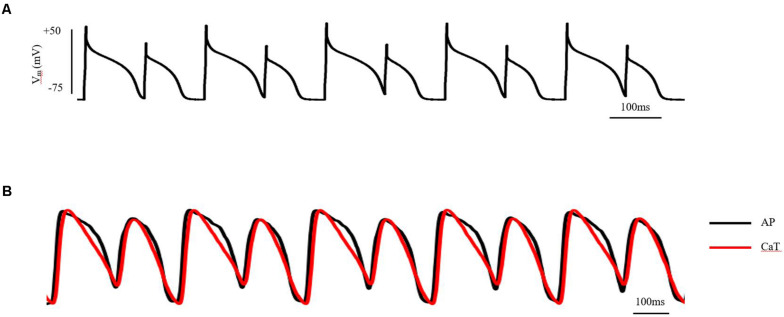
AP and CaT alternans at the cellular and tissue level measured by patch-clamp and optical mapping technology. **(A)** AP alternans of ventricular myocytes in adult guinea pigs, induced by S1S1 stimulation protocol. Stimulus: 1.5-fold threshold; **(B)** AP and CaT alternans of isolated guinea pig heart, induced by S1S1 stimulation protocol. Stimulus: 2-fold threshold, apex. See the Supplementary Material 1 for detailed experimental methods.

Cardiac electrical alternans, first described by Lewis in 1910 (Cherry and Fenton, 2004), is manifested as every-other-beat alternations in the morphology of the electrocardiogram (ECG) observed in data from the same underlying pacing site. Clinically, T-wave alternans (TWA) is the most common form of cardiac electrical alternans. It is associated with the dispersion of repolarization and can be used as a biomarker to predict malignant arrhythmias and sudden cardiac death (SCD; Hekkanen et al., 2020). At the cellular level, TWA is measured as every-other-beat variations in action potential morphology (AP alternans) and/or in the cytosolic calcium transient (CaT alternans) at a constant stimulation frequency. At the tissue level, cellular AP alternans can be reflected as spatially concordant alternans (SCA), where the whole tissue exhibits action potential duration (APD) alternans of in-phase changes. It may also be reflected as spatially discordant alternans (SDA), in which the APD in different regions exhibit APD alternans of out-of-phase changes or otherwise offset (higher period) phase (Munoz et al., 2018; Colman, 2020). Although the whole tissue exhibits APD alternans of in-phase changes when SCA happens, it is a pure electrical mechanism that relies on the non-uniform arrival time of paced excitations, increasing spatial heterogeneity at the same time point (Qu et al., 2000; Huang et al., 2020). By its nature, SDA has the potential to produce larger spatial repolarization dispersion than SCA, thus promoting unidirectional conduction block and leading to reentry. Experimentally, pact-clamp and optical mapping methods can be used to investigate cardiac alternans in AP and in the intracellular Ca^2+^ transient (CaT), as well as the association between the two at the cellular and tissue levels *in vitro* (Chou et al., 2017; Munoz et al., 2018). A schematic diagram of AP and CaT alternans at the cellular and tissue level measured by patch-clamp and optical mapping technology is shown in Figure 1 (detailed experimental methods are shown in the Supplementary Material 1).

In 1948, Kalter and Schwartz (1948) found that TWA visible to the naked eye was associated with reduced survival in five ECG cases of 6059 patients (0.08%). In 1984 and 1988, Adam et al. (1984), Smith et al. (1988) found that analysis of microvolt TWA (MTWA) can provide a non-invasive measurement of cardiac electrical instability by using a novel multidimensional spectral technique. More recently, MTWA evaluated by the spectral method during both exercise and ambulatory ECG monitoring has been extensively studied for risk stratification, especially in aiding the decision-making for cardioverter defibrillators (ICD_s_) implantation. Apart from TWA visible to the naked eye, several technologies to evaluate MTWA based on the ECG have been developed over the last two decades. Only two of these technologies have been studied in sizeable clinical studies, namely, the modified moving average (MMA) method and the spectral method. The MMA method allows for estimation of TWA during both exercise and in ambulatory ECG monitoring of daily activities (Nearing and Verrier, 2002; Burattini et al., 2009). The study of Kraaier et al. (2011) showed that there is a good correlation between these two methods for evaluating and measuring MTWA. Clinically, MTWA is commonly measured using the spectral method, although the MMA method assessment for ambulatory ECG recordings has also been developed. Since the classical spectral method is largely limited by a considerable proportion of non-determined results, more attention is being paid to the MMA method for TWA testing in ambulatory ECG recordings, which does not require achieving a target heart rate or drugs’ washout (Lewek et al., 2017).

Subsequent studies have provided further evidence that TWA is a marker of cardiac electrical instability and SCD in a wide range of clinical settings. Its association with increased risk of cardiac arrhythmogenesis has been reported in multiple heart diseases, such as acute myocardial infarction (Puletti et al., 1980; Giudici and Savage, 1990), heart failure (HF; Luomanmaki et al., 1975; Arsenos et al., 2018), long QT syndromes (LQTS; Shimizu and Antzelevitch, 1999; Gadage, 2018; Kannampuzha et al., 2018), catecholaminergic polymorphic ventricular tachycardia (CPVT; Yang et al., 2016), and polymorphic ventricular tachycardia (Holley and Cooper, 2009). Following the first reports on the great significance of a spectral method in predicting SCD (Klingenheben et al., 2000), ACC/AHA/ESC 2006 guidelines recommended TWA assessment as Class II_a_ in risk stratification of patients with ventricular arrhythmias (Zipes et al., 2006). The International Society for Holter and Non-invasive Electrophysiology released an MTWA expert consensus in 2011, recommending that TWA can be used to predict malignant ventricular arrhythmias and SCD in high-risk patients (Verrier et al., 2011). Positive and negative predictive values of TWA testing is about 50–80% and 75–98%, respectively, depending on the nature of the population being tested (Tsai et al., 2002) and is more accurate in predicting arrhythmia events than other non-invasive measures (Hohnloser et al., 1998). Recently, Pang et al. (2019) compared TWA and heart rate variability in chronic epileptic patients compared to patients with newly diagnosed epilepsy (monitored simultaneously with 14-day Zio XT extended continuous ECG patch monitor). They showed that elevated TWA levels have a high correlation equivalently on ECG patch and Holter recordings in patients with chronic epilepsy across 24 h. However, cardiac electrophysiology has complex spatiotemporal dynamics. It is very challenging to predict the occurrence of arrhythmias and prevent their transition from a stable rhythm to cardiac alternans. As a marker of electrical instability, the study of TWA mechanisms and the mechanism of transition to arrhythmia have become an urgent clinical need.

**FIGURE 2:**
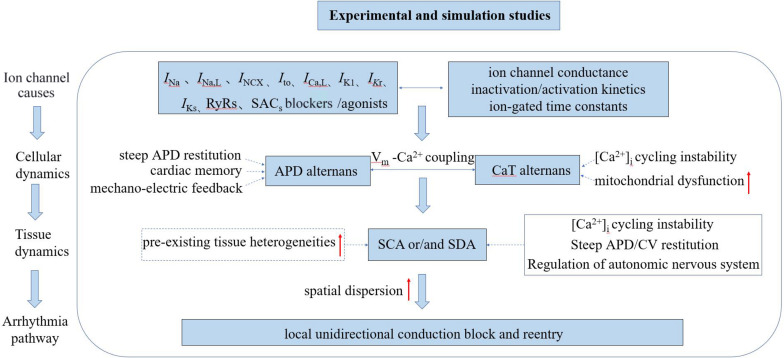
Schematic diagram illustrating the mechanisms of cardiac alternans and arrhythmogenesis associated with experimental and simulation studies. *I*_Na_, inward Na^+^ current; *I*_Na,L_, late sodium current; I_NCX_, sodium-calcium exchanger current; *I*_to_, outward transient potassium current; *I*_Ca,L_, L-type Ca^2+^ current; *I*_K1_, inward rectifier current; *I*_Kr_, rapid delayed rectifier potassium current; *I*_Ks_, slow delayed rectifier potassium current; RyR_s_: ryanodine receptors; SAC_s_, stretch-activated channels; SCA/SDA, spatially concordant/discordant alternans; and CV, conduction velocity.

In this manuscript, we present a more comprehensive review associated with underlying mechanisms of TWA and arrhythmogenesis at both cellular and tissue levels revealed through experimental and simulation studies (the mechanism of TWA have also been reviewed: Surawicz and Fisch (1992), Armoundas and Cohen (1997), Armoundas et al. (2002), Walker and Rosenbaum (2003), Narayan (2006, 2008), Weiss et al. (2006), Wilson and Rosenbaum (2007), Merchant et al. (2013), Qu et al. (2013), Disertori et al. (2016), Colman (2020). One of the most well-known hypotheses for the generation of APD alternans is the APD restitution theory, which was initially established by Nolasco and Dahlen (1968) attributing the generation and persistence of cardiac alternans to the maximum slope of the APD restitution curve. When the maximum slope is greater than one, a small diastolic interval (DI) change will lead to the APD gradually drifting away from the equilibrium point, resulting in continuous and stable alternation. However, owing to the effect of cardiac memory, some other studies (Comlekoglu and Weinberg, 2017; Song and Qu, 2020) have found that the APD restitution theory by itself is insufficient to produce stable alternans but instead involves more complex dynamic processes. Another potential mechanism is related to CaT alternans, which mainly involves the unstable state of calcium content in the sarcoplasmic reticulum (SR; Wang et al., 2014; Sun et al., 2018; Liu et al., 2019) or mitochondrial dysfunction (Oropeza-Almazan and Blatter, 2020). By mechano-electrical coupling, CaT alternans may manifest as APD alternans, and then manifested as TWA on the ECG. As the coupling between membrane voltage (V_m_) and calcium handling dynamics is bi-directional, however, it is hard if not impossible to identify which is the main and the secondary mechanism between the two. Under diseased conditions in which the feedback between membrane potential and intracellular calcium ([Ca^2+^]_i_) is positive, Hopf bifurcations occur, resulting in periodic oscillatory behaviors. When this feedback is negative, period-doubling bifurcation routes to chaos occur (Landaw and Qu, 2019). At the tissue level, APD alternans exhibits temporal and spatial heterogeneity with increasing pacing frequency. As of now, possible mechanisms underlying the pro-arrhythmogenesis of SCA and/or SDA have also been proposed, although it is still incompletely understood. It has been suggested that the increased risk of arrhythmogenesis may result from the amplification of pre-existing tissue heterogeneity by the SCA/SDA-induced functional heterogeneity of cardiac tissue leading to substrates favoring the initiation and maintenance of arrhythmias (Tse et al., 2016a). Under rapid pacing or pathological conditions, it can be envisaged that tissue’s intrinsic electrophysiological heterogeneities will be further amplified, resulting in an increased spatial dispersion in cardiac excitation/recovery leading to unidirectional or localized conduction block forming substrates for arrhythmias. However, some experimental and simulation studies have shown that pre-existing tissue heterogeneities may not be necessary for the formation of SDA preceding to arrhythmogenesis (Watanabe et al., 2001; Huang et al., 2020). It is known that many dynamic properties, such as conduction velocity (CV) restitution (Wei et al., 2015; Wang et al., 2018; Huang et al., 2020), steep APD restitution curve (Qu et al., 2000; Liu et al., 2015; Vandersickel et al., 2016), [Ca^2+^]_i_ cycling instability (Alvarez-Lacalle et al., 2015; Qu et al., 2016; Restrepo et al., 2018; Song et al., 2018), and autonomic nervous system regulation (Lown and Verrier, 1976; Winter et al., 2018; Xiong et al., 2018) can convert SCA into SDA facilitating arrhythmogenesis. The schematic diagram illustrating the mechanisms of cardiac alternans and arrhythmogenesis associated with experimental and simulation studies is shown in Figure 2.

From the literature search, the number of publications relating to TWA is declining; the subject is becoming less popular. One of the reasons for this might be that the original hope that TWA could help in clinical risk stratification is fading away. Importantly, although possible mechanisms of TWA have been investigated in previous studies (Qu et al., 2000; Restrepo et al., 2018; Wang et al., 2018; Xiong et al., 2018; Huang et al., 2020), the spontaneous transition from spatially concordant or discordant alternans to arrhythmias is yet to be understood. Given the fact that TWA is associated with arrhythmogenesis, it is of importance to reveal such mechanisms by using both experimental and computational modeling approaches, the results of which may provide critical information that is useful for arrhythmia prevention, diagnosis, and drug intervention. Based on this, we aim to present up to date progress in the study of electrophysiological mechanisms of TWA by both experimental and computer simulation approaches.

## Mechanisms of Cardiac Alternans and Arrhythmogenesis

T-wave alternans is the most general form of cardiac electrical alternans and the pathophysiological mechanism has only been partially elucidated. The underlying electrophysiological basis for TWA was initially thought to be alternating conduction pathways, originating from regional areas of refractoriness that every-other-beat alternated spatially (Smith and Cohen, 1984). Transmural-ventricular repolarization dispersion is an existing general electrophysiological phenomenon, but this spatiotemporal dispersion of myocardial repolarization is certainly enhanced by alternans (especially by SDA), which is the electrophysiological basis for TWA (Kleinfeld et al., 1963). In 1999, Pastore et al. (1999) recorded by optical mapping action potentials from 128 sites on the epicardial surface of guinea pig hearts during ventricular pacing. Their results suggested alternation of the repolarization phases of the AP associated with the alternation in the T wave on the ECG as heart rate increased. High temporal and spatial resolution optical mapping has since been developed, and the voltage and calcium recordings were synchronized using a high-resolution (128 × 128 pixels, 900 frames/s) optical mapping system based on a CMOS camera in our experiment (a schematic diagram of voltage/Ca signal of a single-pixel, APD_80_ map, CaT amplitude map, and activation times map is shown in the Supplementary Material 2). So far, subsequent experiments and clinical evidence further suggest that the electrophysiological basis of TWA is associated with the alternation of repolarization in cardiomyocytes (Wang et al., 2018; Colman, 2020). Repolarization alternans at the cardiomyocyte level is several orders of magnitude larger than the corresponding magnitude of TWA, which can explain why even microvolt-level TWA detected by the ECG may be physiologically and clinically critical (Walker and Rosenbaum, 2003). Therefore, TWA is associated with every-other-beat alternations of the repolarization at the cellular level and the bifurcations often get bigger and then smaller with increased pacing rate, recombining at faster rates. It is important to determine the underlying cellular and subcellular mechanisms for alternans as presumably an abnormality in cellular and subcellular can contribute to the pathophysiology of malignant arrhythmias and SCD at the tissue and whole organ levels. Herein, we review the mechanisms of cardiac alternans and arrhythmogenesis associated with the simulation and experimental data.

### Cellular Mechanisms of Cardiac Alternans

#### Alternans Due to a Steep APD Restitution Curve

##### General description of the APD restitution theory

**FIGURE 3:**
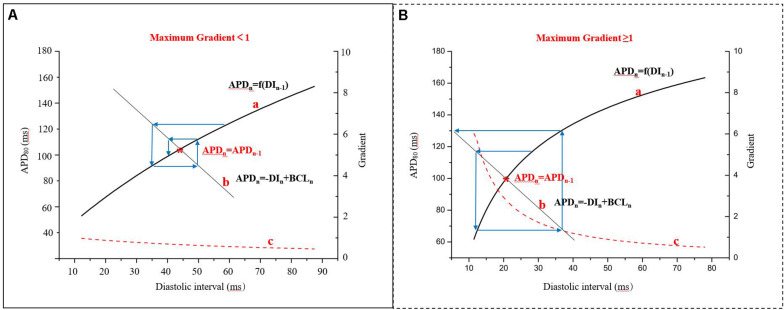
Cobweb plots used to indicate the stability of APD alternans. Curve a: APD restitution curve, APD_*n* + 1_ = *f*(*DI*_*n*_); curve b: the fixed relationship of BCL, APD, and diastolic interval (DI), BCL = APD + DI; curve c: the gradient of the APD restitution curve; red star: the equilibrium point (APD_n_ = APD_n__–__1_), that is the interaction point of the curve a and curve b (modified from Nolasco and Dahlen, 1968). **(A)** The maximum gradient is less than one and **(B)** The maximum gradient is greater than or equal to one.

In 1968, the mechanism of alternans was first described using a graphical method, relating them to APD restitution (Nolasco and Dahlen, 1968). This refers to the normal shortening of the APD in response to a faster heart rate and is considered an adaptive mechanism to preserve diastole at this rate. It is defined as the dependence of the APD on the previous DI, APD_*n* + 1_ = *f*(*DI*_*n*_). The kinetics of restitution are described by the restitution curve, a plot of APD vs. previous DI. As shown in the Supplementary Material 3, it can be obtained by using a standard stimulation (S1S2) protocol (an extra stimulus application at different coupling intervals after a series of regular pacing pulses) or by using a dynamic pacing protocol (S1S1; steady pacing at gradually increasing rates). While both protocols have been used to measure APD restitution, the restitution curve in standard stimulations is a measure of the immediate response to basic cycle length (BCL) changes, whereas the dynamic restitution curve is a measure of the steady-state response (Jing et al., 2015; Osadchii, 2019). Previous studies have indicated that steady-state alternans may be better described by APD adaptation dynamics, depending on both the steady-state relationship between the BCL and APD and the restitution curve (Osadchii, 2019). The stability of AP alternans can be shown by the cobweb plots (Figure 3). If the maximum gradient is less than one (Figure 3A), the alternans are transient and will return to the stable equilibrium point (Figure 3, red star) over subsequent beats. When the maximum gradient of the APD restitution curve is greater than or equal to one (Figure 3B), stable AP alternans and other complex dynamics will occur (Nolasco and Dahlen, 1968). For each BCL, there is a steady-state or equilibrium point. Graphically, due to the fixed relationship (BCL = APD_*n*_ + DI_*n*_), longer DI allows for channels to fully recover by the next beat, resulting in longer APD, and the cycle of alternation begins.

##### The individual role of ion channels in contribution to the APD restitution curve

The gradient of the APD restitution curve (Figure 3 curve c) is a collective measure of the recovery of all ion channels opened during the time course of cardiac APs. AP alternans occurs when the heart rate exceeds the ability of certain ion channels involved in the repolarization to completely recover from activation or inactivation depending on their time-dependent gating kinetics. Firstly, normal Na^+^ channels are inactivated mainly during the plateau of the AP and they have lesser direct effects on the APD, but incomplete recovery of these Na^+^ channels lowers the amplitude of AP which impacts the activation of other ion channels, influencing APD. The recovery of Na^+^ channels, for example, slows down under ischemic conditions (Kang et al., 2020), and their effects on APD recovery will be expanded to longer DI (>40 ms). In a dog HF model, the late sodium current (*I*_Na,L_) blocker ranolazine attenuated arrhythmogenic cardiac alternans by suppressing CaT alternans and decreasing the Ca^2+^-voltage coupling gain (Fukaya et al., 2019). Recently, Wang et al. (2018) have put forward the potential role of impaired Na^+^ channel activity in contributing to the transition from AP alternans in cardiomyocytes to arrhythmias, using multiscale rabbit ventricular models. Secondly, the L-type Ca^2+^ channel recovers more slowly than the Na^+^ channel, which has a major impact on the APD restitution slope due to the occurrence of a major part of the inward current during the plateau phase. The time-dependent recovery from inactivation of L-type Ca^2+^ channels has been hypothesized to be a causative factor for the generation of AP alternans in all ion channels during the cardiac AP (Kanaporis and Blatter, 2017). Inhibition of these L-type Ca^2+^ leads to reduced gradients of the APD restitution curves (Sun et al., 2018). In simulations, an increase in the magnitude of the inward Na^+^ current (*I*_Na_; Qu et al., 1999, 2004) and a reduction in the time constant of the inactivation gating variable for the L-type Ca^2+^ current (*I*_Ca,L_; Qu et al., 1999; Fox et al., 2002b) can reduce alternans amplitude. Thirdly, time-dependent K^+^ channels, especially the voltage-gated delayed rectifiers, show the slowest recovery compared to other ion channels. Experimental results show that increasing the amplitudes of the inward rectifier current (*I*_K1_) and the two components of the delayed rectifier potassium current (*I*_Kr_ and *I*_Ks_) will suppress alternans by reducing the APD and prolonging the DI (Tachibana et al., 1999). In simulations, Fox et al. (2002b) also showed that APD alternans was suppressed by increasing the amplitude of *I*_K1_, *I*_Kr_, or *I*_Ks_. For other ion channels, the APD alternans resolved when the outward transient potassium current (*I*_to_) was blocked, suggesting that the alternans was associated with *I*_to_. *I*_to_-mediated spike-and-dome AP morphology is linked to Brugada syndrome (BrS). TWA is widely observed in BrS and alternans occurs at normal heart rates in which the DI is still very long (Ni et al., 2019). A possible mechanism of BrS based on experiments and computer simulations (Miyoshi et al., 2005) indicates that dynamical instabilities that can cause alternans and other complex AP dynamics also promote dynamic substrates at the tissue level that, differently from static heterogeneity, can potentiate phase-2 reentry. Therefore, the gradient of the APD restitution curve may be flattened by changing the kinetics of one or more membrane currents, and this has been shown to prevent the development of SDA and arrhythmogenesis (Qu et al., 2000). It is still currently unclear whether these ion channels represent a primary or a secondary mechanism for alternans.

##### Cardiac memory

Some inconsistent reports have been published of the effect of cardiac memory. In some studies, it has been indicated that APD alternans is seen for a shallow APD restitution curve (maximum gradient < 1) or no APD alternans is observed for a steep curve (maximum gradient > 1; Ponnaluri et al., 2016; Vandersickel et al., 2016). In 1982, Rosenbaum et al. (1982) suggested that changes in electrotonically modulated T waves show memory and accumulation, which may last for days or weeks after the provoking stimulus is discontinued. APD depends not only on the immediately preceding DI but also on the series of DI preceding it, *APD*_*n* + 1_ = *f*(*DI*_*n*_ + *DI*_*n*−1_ + …), due to the slow recovery of ion channels and the gradual accumulation of ions on both sides of the cell membrane. The short-term memory effect is called APD accommodation (Zhang et al., 2011). Moreover, rate-dependent memory which has been shown to contribute to repolarization alternans observed in humans is called hysteresis. This refers to alternans caused by rapid pacing, which persist despite a subsequent slowing of heart rate (Burattini et al., 2016). Due to the slow APD adaptation to BCL change, it may take many seconds for the APD to reach its steady-state value. As BCL shortens, APD becomes shorter and the APD restitution curve becomes less steep than it was initially. The presence of cardiac memory suggests that the pacing protocol is important, so studies of alternans occurrence should specify initial pacing conditions. In an isolated rabbit heart experiment, the effect of short-term memory was estimated by calculating the τ (APD = APD_0_ + *a* × *exp*^(−*t*/τ)^, time constant) of APD accommodation, and results showed that the mechanism associated with short-term memory can also be the basis for cardiac alternans formation in the heart (Wei et al., 2015). Kalb et al. (2004) presented a new method to investigate the rate- and memory-dependent aspects of APD restitution, showing that none of these gradients approached unity for the persistent 2:2 response (alternans), and demonstrating that the gradient of the traditional restitution curve cannot be used to accurately predict the steady-state alternans. In their simulation, the gradient of the APD restitution curve alone is not an accurate parameter to predict alternans, which has been confirmed by mathematical models (Comlekoglu and Weinberg, 2017; Song and Qu, 2020). The effective refractory period (ERP) restitution (which is easier to measure than APD restitution in clinical settings) was also found to be a reliable indicator of alternans in the Courtemanche et al. human atrial cell model (Xie et al., 2002) and ventricular myocytes experiments during hypokalemia (Osadchii et al., 2010). Furthermore, the gradient of APD restitution may fail to predict alternans when [Ca^2+^]_i_ cycling has a strong influence on AP.

#### Alternans Due to the Bi-Directional Coupling Between Voltage and [Ca^2+^]_i_

In cardiomyocytes, the beat-to-beat regulation of membrane potential and cytosolic calcium is coupled and involves complicated feedback mechanisms. On the one hand, Ca^2+^ → voltage coupling means that a larger CaT can enhance the sodium-calcium exchanger current (*I*_NCX_) to prolong the APD by increasing net inward current produced during the plateau of AP, but on the other hand it can also facilitate the inactivation of *I*_Ca,L_ by Ca^2+^-dependent inactivation and thus shorten the APD. Other calcium-sensitive currents in small quantities, such as calcium-activated non-selective cation currents and calcium-activated chlorine currents, can also affect the membrane voltage (Kanaporis and Blatter, 2016). It is generally accepted that this relationship represents a key cause for electromechanical and CaT alternans (Weiss et al., 2006). Based on the fixed relationship (BCL = APD_*n*_ + DI_*n*_), a longer DI allows more time for recovery of *I*_Ca,L_, leading to enhanced *I*_Ca,L_, larger Ca^2+^ release, and longer APD during the following beat. In l971, Spear and Moore (Spear and Moore, 1971) were the first to describe this in a highly hypothetical paper, suggesting that the APD might be controlled by intracellular free calcium, based on simultaneous recordings of AP and contraction alternans. However, voltage → Ca^2+^coupling means that [Ca^2+^]_i_ will also alternate in response to the alternating amplitude of *I*_Ca,L_ when APD alternates. A longer DI allows for a longer recovery time for L-type calcium channels, which would increase the SR Ca^2+^ load and therefore the CaT amplitude. If APD alternans is a consequence of electrical instability, the CaT will also alternate by excitation-contraction coupling. Therefore, the potential interdependence of [Ca^2+^]_i_ cycling and membrane voltage presents a difficult “chicken and egg” problem that remains unresolved as experiments cannot effectively quantify their respective roles as of yet.

#### Alternans Due to Instabilities of [Ca^2+^]_i_ Handling

It has been known for several decades that APD alternans is suppressed or inhibited by verapamil (Marcantoni et al., 2019), caffeine (Wang et al., 2014), BayK8644 (Saitoh et al., 1988), nisoldipine (Saitoh et al., 1988), and ryanodine (Sun et al., 2018), all of which point to a mechanism related to [Ca^2+^]_i_ handling. Substantial follow-up studies have suggested that disturbances in every-other-beat [Ca^2+^]_i_ cycling constitute the main cause of AP alternans (Picht et al., 2006; Wang et al., 2014; Sun et al., 2018; Liu et al., 2019; Oropeza-Almazan and Blatter, 2020). From a subcellular perspective, the force generated during cardiac contraction is formed by the interaction between myosin and actin proteins and the cross-bridge cycling in thousands of sarcomeres per cardiomyocyte. For each myocyte, this electrical depolarizing signal is converted to a chemical signal, namely an increase in free [Ca^2+^]_i_ concentration. To maintain blood circulation in the heart, individual sarcomeres have to contract in the synchronous pattern. Loss of temporal organization of this process can cause circulatory arrest, for example, in the early phase of VF.

It may therefore be helpful to review the key processes involved in the uptake, buffering, and release of [Ca^2+^]_i_ before discussing the specific role of [Ca^2+^]_i_ handling in the mechanism of alternans. Briefly, activation of *I*_Ca,L_ by the depolarizing wavefront causes free Ca^2+^ to cross the membrane close to the T-tubule, and the cytoplasmic Ca^2+^ binds to the ryanodine receptors (RyRs) of the SR nearly synchronously, which releases abundant Ca^2+^ stored in the SR into the cytosol through Ca-induced Ca-release mechanisms (Tse et al., 2016a). When *I*_Ca–L_ is blocked, or in the case of prolonged depolarization, the release of SR Ca^2+^ can also be stimulated by inward Ca^2+^ current from the Na^+^-Ca^2+^ exchanger (NCX) working in “reverse mode.” After each contraction, the majority of cytoplasmic Ca^2+^ are taken back into the SR by SR-Ca-ATPase (SERCA) and, to a lesser extent, by NCX working in “forward mode.” In this case, cell V_m_ is determined by the consequences of [Ca^2+^]_i_ dynamics, Ca^2+^-dependent ion currents, and Ca^2+^ fluxes and transporters (the instabilities of intracellular Ca cycling in alternans is discussed in detail below: section “SDA and arrhythmogenesis arising from the instabilities of [Ca^2+^]_i_ handling”).

#### Alternans Due to Mechano-Electric Feedback

The heart is an electrically driven mechanical pump. Interestingly, the heart can translate mechanical stimuli into electrical signals through mechano-gated ion channels, and mechanical contraction can regulate the electrical properties of the myocytes. This phenomenon referred to as mechano-electric feedback (MEF; Hazim et al., 2019). Experimentally, electrical alternans has been observed during simulated pulsus alternans produced by clamping the proximal aorta on alternate beats in a pig model of myocardial ischemia (Murphy et al., 1996), suggesting that alternating stretching of the ischemic segment during alternating pulsus contributes to electrical alternans. In this case, the myocytes of the vessel wall did not contract and changes in [Ca^2+^]_i_ probably did not cause electrical alternans. Rather it was the mechano-sensitive ion channels, such as volume- or stretch-activated channels (SACs), that were responsible. Indeed, these mechano-sensitive ion channels are also able to affect membrane potential on an every-other-beat basis and therefore may influence cardiac dynamics. Generally, the effects of MEF on the electrophysiology at high pacing rates are shown to be proarrhythmic (Hazim et al., 2020).

### Mechanisms of Alternation and Arrhythmogenesis at Tissue-Level

**FIGURE 4:**
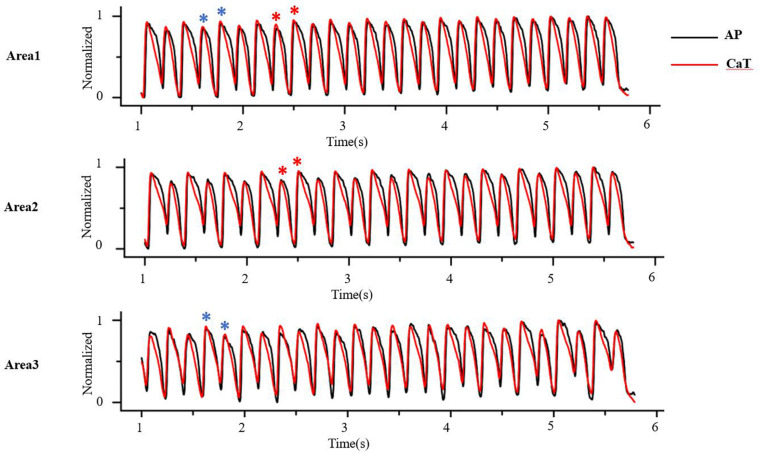
At the tissue level, cellular alternans be manifested as spatially concordant and/or discordant alternans. As the red star shows, AP and CaT alternans is in-phase changes in whole tissue, that is, spatially concordant alternans (SCA); as the blue star shows, AP and CaT alternans is out-of-phase changes in different regions, that is, spatially discordant alternans (SDA) (Optical mapping in isolated guinea pig heart by S1S1 stimulation).

At the tissue level, cellular alternans can be reflected as SCA, in which the whole tissue exhibits AP and CaT alternans of in-phase changes (a schematic diagram of SCA is shown in Figure 4, red star) and/or SDA, in which different regions exhibit AP and CaT alternans of out-of-phase changes (A schematic diagram of SDA is shown in Figure 4, blue star) or otherwise offset (higher period) phase (Colman, 2020). APD alternans, whether SCA or SDA, can produce arrhythmias. For example, SCA themselves can produce a 2:1 conduction block, thereby initiating reentry (Fox et al., 2002a; Tse et al., 2016a). It is a pure voltage mechanism that relies on the non-uniform time of arrival of paced excitations. By its nature, SDA has the potential to produce larger spatial repolarization dispersion, and is therefore more likely to promote unidirectional conduction block and leading to reentry (Wang et al., 2014, 2018; Liu et al., 2015; Wei et al., 2015; Huang et al., 2020). In theory, simulation of the electrical wave propagation in the heart can be used to clarify the mechanisms of reentrant arrhythmia and develop new treatment strategies. Deng et al. (2019) simulated ventricular tachycardia using ventricular models reconstructed from late gadolinium-enhanced magnetic resonance imaging. They investigated the effects of electrophysiological parameters on ventricular tachycardia and predicted ablation targets in the heart model. In clinical trials, technologies available for mapping action potentials from multiple sites on an isolated heart was formerly limited to monophasic action potential electrodes and arrays of electrograms (EGMs). Currently, several mechanisms have been identified to be responsible for the formation of SDA and arrhythmogenesis based on the development of theories and experiments, including (1) dynamic factors, such as CV restitution with steep APD restitution curve or [Ca^2+^]_i_ cycling instability; Echebarria and Karma (2002) derive an equation that governs the spatiotemporal dynamics in paced cardiac tissue and showed that a pattern-forming linear instability causes the spontaneous formation of traveling or stationary waves whose nodes divide the tissue into regions with the opposite phase of APD alternans; (2) pre-existing tissue heterogeneities, including structural (e.g., cellular electrical coupling, structural barrier) and electrophysiological (e.g., the endo-epi distribution of APD and [Ca^2+^]_i_ cycling heterogeneities); and (3) regulation of the autonomic nervous system.

#### SDA and Arrhythmogenesis Arising From CV Restitution

##### General description of CV restitution

Conduction velocity restitution has been shown to affect the dynamics of impulse propagation and the stability of spiral wave reentry. Steep CV restitution, i.e., when the CV of a propagating wave is steeply dependent on the preceding DI, is a key dynamical factor in promoting SDA (Wei et al., 2015; Wang et al., 2018; Huang et al., 2020). Logically, any intervention that causes significant gradients of the DI has the potential to induce SDA, based on the principles of restitution. At high pacing frequencies, there is a sufficiently short DI behind a long APD due to the adaptive mechanism of the heart, resulting in some ion channels not fully recovering from inactivation. Based on CV restitution, the CV slows down on the subsequent beat. Conversely, slower conduction increases the DI slightly which allows the APD to increase slightly. This process is gradually amplified in subsequent beats, eventually resulting in SDA. If APD alternans occurs in a DI range in which the CV is not also changing, alternans is spatially concordant in tissue. However, if APD alternans occurs in the DI range in which the CV is also varying with DI (at short DI), then SDA can form if the tissue size is adequate. Weiss et al. (1999) summarized evidence that indicates that the destabilizing oscillatory motions during spiral-wave reentry is caused by the restitution properties of the APD and CV in the simulations of 2-dimensional model of cardiac tissue. Since the QT interval and the T wave in the ECG correlate with the APD and its spatial distribution, APD alternans is manifested as TWA in ECG. CV varies during SDA, which affects the width and amplitude of QRS in ECG, resulting in both T-wave and QRS alternans. This prediction agrees with the experimental observation that TWA only occurred during SCA, but both QRS and TWA occur during SDA (Qu et al., 2010).

##### Experimental findings of AP conduction during alternans

Conduction velocity restitution as a mechanism of SDA was first reported by Cao et al. (1999) who performed computerized epicardial mapping studies in dogs and used the Luo-Rudy I ventricular AP model to reproduce experimental results. Their findings supported a key role for both CV and ERP restitution in the initiation of VF by rapid pacing. Mironov et al. (2008) investigated the mechanisms underlying SDA development in isolated rabbit hearts, finding a correlation between steep CV restitution and small τ (time constant) and stable nodal lines. Such correlation depends almost entirely on the recovery of sodium channels because the depolarization of the AP is primarily determined by these channels. As mentioned earlier, sodium channels can recover rapidly from inactivation under normal conditions. However, if the recovery of sodium channels slows down under pathological conditions, their impact on CV restitution will expand to longer DI, resulting in SDA even at normal or slow heart rates, thus increasing the risk of arrhythmias (Wang et al., 2018). Therefore, this mechanism may be particularly important under ischemia or sodium channel blockade, where sodium channel recovery slows down, causing post-repolarization refractoriness (Qu et al., 2004).

##### Simulation findings of AP conduction during alternans

In simulation studies, numerous reports have also suggested that CV restitution is a key dynamic factor leading to SDA and arrhythmogenesis in cardiac tissue. Watanabe et al. (2001) demonstrated that SDA could form spontaneously through the interaction of CV and APD restitution at high pacing frequencies or through the dispersion of DI produced by ectopic foci, utilizing one- and two-dimensional simulation of AP propagation models. Qu et al. (2000) also explained the SDA arises dynamically from APD and CV restitution properties and markedly increases the dispersion of refractoriness, through a two-dimensional tissue model based on the Luo-Rudy I cardiac AP model. Fox et al. (2002c) suggested that rapid periodic pacing induced marked spatiotemporal heterogeneity of cellular electrical properties, culminating in paroxysmal conduction block. These behaviors resulted from a non-uniform distribution of APD alternans, secondary to CV alternans. Whether pre-existing spatial heterogeneity is the cause of SDA formation under the premise of CV restitution is still under discussion. Some results have demonstrated that SDA can develop without spatial tissue heterogeneities in electrophysiology due to the characteristics of CV restitution (Qu et al., 2000; Hayashi et al., 2007). In any case, some studies have suggested that both tissue heterogeneity and steep CV restitution can be involved in the formation of SDA (Qu et al., 2000); in their work Huang et al. (2020) showed both SCA and SDA in tissues without initial spatial tissue heterogeneities in electrophysiology due to the characteristics of CV restitution. When CV restitution is not engaged, the APD spatial pattern in tissues can have multiple solutions, including SCA and different SDA patterns, depending on initial conditions or pre-existing repolarization heterogeneities.

#### SDA and Arrhythmogenesis Arising From the Steep APD Restitution Curve

The cellular mechanism of alternans caused by a steep APD restitution curve has been mentioned earlier, but a steep APD restitution curve can further convert SCA to SDA at the tissue level (Liu et al., 2015). In restitution curves with a slope greater than one at short DI, APD alternans and other complex dynamics can occur, especially [Ca^2+^]_i_ cycling dynamics. In this case, as BCL decreases, bifurcations from the stable equilibrium state (1:1) to alternans (2:2) and from 2:2 alternans to 2:1 block can be observed, and more complex dynamics at even faster pacing rates. Conrath and Opthof (2006) have critically reviewed some mechanistic aspects of the genesis of TWA associated with the APD restitution theory. It suggested that SDA results in large spatial gradients of repolarization that can cause local unidirectional conduction block and reentrant ventricular fibrillation (VF; Qu et al., 2000; Liu et al., 2015). It seems to suggest that a reduction of dynamically induced dispersion by appropriate alteration of electrical restitution is a promising antiarrhythmic strategy. In addition to its arrhythmogenic effects at fast heart rates, APD alternans may be applicable to arrhythmogenesis when the APD is substantially prolonged, such as in LQTS or HF, so that the DI becomes short enough to engage the steep slope range of the APD restitution curve, even at normal heart rates. However, there are some conflicting reports observed that an increase in arrhythmogenic potential is not due to the steepening of the APD restitution curve. Its onset is determined by the global properties of the APD-restitution curve and not by its slope (Vandersickel et al., 2016) or by a novel blocking mechanism in a mathmatical model of HF (Ponnaluri et al., 2016).

##### Experimental evidence to support the APD restitution theory

After Nolasco and Dahlen’s important early study (Nolasco and Dahlen, 1968), the APD restitution theory has been further validated by some experimental studies. The restitution kinetics of APD have been investigated in isolated, perfused, canine left ventricles during VF with standard (S1S2) stimulation and dynamic pacing (S1S1) protocol. This has provided further evidence for a strong relationship between the kinetics of electrical restitution, the occurrence of APD alternans, and complex APD dynamics during VF (Koller et al., 1998). In the canine ventricle, Riccio et al. (1999) have shown that drugs that reduce the slope of the restitution curve (diacetyl monoxime and verapamil) prevent the induction of VF and convert existing VF into periodic rhythms. Conversely, a drug that does not reduce the slope of the restitution curve (procainamide) does not prevent the induction of VF. Garfinkel et al. (2000) have also shown that bretylium acts in accord with the restitution hypothesis: flattening the restitution curve prevents wave breaking and thus prevents VF in farm pigs. They have suggested that flattening the slope of the restitution relation and thereby decreasing the magnitude of APD alternans has been shown to have an antifibrillatory effect.

##### Modeling studies to support the APD restitution theory

This restitution theory has also been further validated by modeling studies. Using a kinematic model to represent wavefront-waveback interactions and simulations with the Luo-Rudy model in a one-dimensional cable of cardiac cells, Qu et al. (2006) suggested that steep APD restitution promote both SDA, a setting which is particularly prone to conduction block and dispersion of refractoriness in response to multiple extrasystoles, and thus enhance vulnerability to conduction block generally. Wu et al. (2004) have successfully reproduced the major features of type 2 VF (showing local spatiotemporal periodicity) by using the Luo-Rudy AP model in a simulated, 3-dimensional tissue slab, under conditions of reduced excitability and flat APD restitution. Franz also critically discussed the relevance of steep vs flat restitution curves in relation to arrhythmogenesis (Franz, 2003). ten Tusscher and Panfilov (2006) have developed a human ventricular cell model, and suggested that the onset of electrical instability [maximum restitution slopes exceeding 1 (up to 1.5)] is due to the recovery dynamics of the fast sodium current. They concluded that steep APD restitution-mediated electrical instability is a possible mechanism for VF in the human heart. However, Ponnaluri et al. (2016) have instead suggested that arrhythmia is not due to the slope of the APD restitution curve, but rather due to increased gap junction lateralization and lower CV using a multiscale rabbit model of HF. Vandersickel et al. (2016) have also shown that the global alternans instability, closely related to the discordant alternans instability, is determined by the global properties of the APD restitution curve and not by its slope.

#### SDA and Arrhythmogenesis Arising From the Instabilities of [Ca^2+^]_i_ Handling

Release of Ca^2+^ from SR into myocyte cytoplasm and their binding to troponin C is the final signal in myocardial contraction. Synchronous contraction of ventricular myocytes is critical for efficient cardiac pumping function. This requires both shuttling of Ca^2+^ between the cytoplasm and SR in individual myocytes, and myocardial layer synchronization of this process by electrical coupling among ventricular myocytes. Therefore, the instabilities of [Ca^2+^]_i_ handling causes SDA and arrhythmias under diseased conditions, such as CPVT and diabetes (Chou et al., 2017). These mechanisms involve dynamic spatial heterogeneity of myocardial [Ca^2+^]_i_ handling (discussion below: section “SDA and arrhythmogenesis arising from pre-existing tissue heterogeneities”), the bidirectional coupling of [Ca^2+^]_i_ and V_m_, and more complex instabilities of intracellular Ca^2+^ handling.

##### Bidirectional coupling of [Ca^2+^]_i_ and V_m_

[Ca^2+^]_i_ → V_m_ coupling is determined by the fact that [Ca^2+^]_i_ feeds back on V_m_. If the coupling is strong, such that large-amplitude CaT alternans results in large amplitude APD alternans and thus larger dispersion of refractoriness, the SDA and arrhythmogenic circumstances are potentiated. It has been reported that if Ca^2+^ → voltage coupling changed from positive to negative, it will lead to SDA (Sato et al., 2006). And there is evidence that SDA can occur despite positive Ca^2+^ → voltage coupling (Sato et al., 2013). Sato et al. (2006) have reported a novel instability mechanism in which the bidirectional coupling of [Ca^2+^]_i_ and V_m_ can drive the CaT of two neighboring cells to be out of phase, which is then manifested in cardiac tissue by the dynamical formation of SDA. Another possible mechanism associated with Ca-driven alternans to arrhythmias follows from the observation that alternans tends to occur under SR leaks, or with Ca overload, which are the same conditions when Ca waves occur. Simulation (Cui et al., 2009; Rovetti et al., 2010) and experimental (Diaz et al., 2002; Cui et al., 2009) studies have observed that Ca waves occur during alternans, which demonstrates that Ca alternans and Ca waves are linked to each other. Therefore, the association of TWA with arrhythmias under these conditions may not be causal but instead reflect a predisposition to Ca waves which induce delayed after-depolarization in cardiac excitation. V_m_ → [Ca^2+^]_i_ coupling is generally believed to be positive, i.e., a long APD is paralleled by a large amplitude CaT and a strong contraction, and that V_m_-driven alternans is determined by single parameter-APD restitution.

##### Experimental studies of the instabilities of [Ca^2+^]_i_ handling

Several mechanisms of the instabilities of [Ca^2+^]_i_ handling have been described. The foremost mechanism, reported by Wang et al. (2014), supposes that CaT alternans is due to a steep non-linear dependence of SR Ca release upon the diastolic SR Ca load immediately preceding the release. This mechanism means that SR Ca release alternates incidentally with diastolic SR Ca load and leads to APD alternans. When at high pacing frequencies or under certain pathological conditions, impairment in the kinetics of any component of [Ca^2+^]_i_ cycling (such as RyRs, SERCA) can create a situation where cytoplasmic Ca^2+^ cannot be fully reclaimed during each beat. Liu et al. (2019) induced CaT alternans by using an impaired SERCA under ischemic conditions. They have suggested that the “threshold” of the ratio between the diastolic network SR Ca content and the cytoplasmic Ca content can better explain the comprehensive effects of the Ca^2+^ uptake and the Ca^2+^ release on CaT alternans onset. However, Sun et al. (2018) demonstrated that enhanced cardiac RyRs function inhibits CaT alternans in the absence of spontaneous Ca^2+^ release, and that RyRs rather than SERCA, is a key determinant of CaT alternans in intact working hearts. These experiments suggested that the alternation of systolic Ca^2+^ level depends on the alternation of SR Ca^2+^ content. On the contrary, later experiments have since shown that SR Ca^2+^ content remains constant before the occurrence of CaT alternans under some conditions, such as slow recovery from inactivation of RyRs and increased mitochondrial dysfunction. Picht et al. (2006) used direct continuous measurement of intra-SR free [Ca^2+^] ([Ca^2+^]_SR_; using Fluo5N) during frequency-dependent CaT alternans in rabbit ventricular myocytes. Their measurements show that alternations in [Ca^2+^]_SR_ are not required for CaT alternans to occur and it is rather likely that some other factor, such as the slow recovery from inactivation of RyRs, is of greater importance in initiating frequency-induced CaT alternans. Furthermore, the tendency of pacing-induced CaT alternans increases with increasing mitochondrial dysfunction due to inhibition of ATP synthesis or dissipation of the mitochondrial membrane potential (Oropeza-Almazan and Blatter, 2020). Huser et al. (2000) proposed that the alternans that they observed in cat myocytes was caused by a functional coupling between the excitation-contraction coupling and glycolysis. They suggested that the efficiency of excitation-contraction coupling is locally controlled by mechanisms utilizing ATP in the microenvironment of the SR Ca release sites, produced by glycolytic enzymes closely related to the release channel.

##### Simulation studies of the instabilities of [Ca^2+^]_i_ handling

**FIGURE 5:**
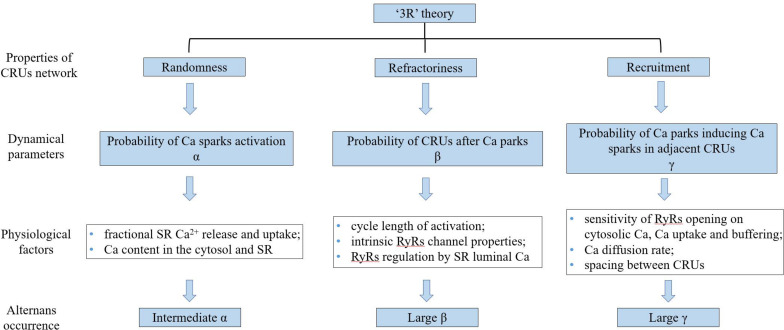
The schematic diagram of “3R” theory. “3R” theory, in which Ca alternans emerges as collective behavior of Ca sparks, determined by three critical properties of the CRU network from which Ca sparks arise. CRU_s_: Ca release units, SR: sarcoplasmic reticulum, and RyR_s_: ryanodine receptors.

Theoretical studies (Tao et al., 2008; Weinberg, 2016) have provided evidence supporting that the alternation of systolic Ca^2+^ level depends on alternation of SR Ca^2+^ content. Rovetti et al. (2010) developed a computational model of Ca^2+^ cycling which is composed of a network of coupled Ca release units (CRUs) which suggested that CaT alternans continues even if the SR Ca content is held constant. They proposed a novel theory, the “3R” theory, in which CaT alternans emerges as collective behavior of Ca sparks, determined by three critical properties of the CRU network from which Ca sparks arise: randomness (of Ca spark activation, assume their probability to be α), refractoriness (of a CRU after a Ca spark, β), and recruitment (Ca sparks inducing Ca sparks in adjacent CRUs, assume the recruitment probability to be γ; Cui et al., 2009; Rovetti et al., 2010). Theoretical studies have concluded that alternans occurs in an intermediate range of α, large γ, and large β, which has also been validated in experiments (Qu et al., 2013). α, β, and γ are dynamical parameters that are determined by many physiological factors. For example, α is determined by the properties of the L-type calcium channels and RyRs (Cantalapiedra et al., 2017), such as the open probability and their conductance, which are also affected by the Ca content in the cytosol and SR. β is determined by the cycle length of activation and the CRU refractory properties. The refractoriness of a CRU can be attributed to either intrinsic RyRs channel properties or RyRs regulation by SR luminal Ca, such as by calsequestrin binding to the RyR protein complex. In a spatial Ca cycling model of ventricular myocytes, Qu et al. (2016) demonstrated two mechanisms of CaT alternans: one mainly depends on fractional SR Ca release and uptake, and the other on the refractoriness and other properties of Ca sparks. They also developed an iterated map model, which unifies the two mechanisms into a single cohesive mathematical framework to explain the seemingly contradictory experimental results. Using a mathematical model of calcium cycling, Restrepo et al. (2018) suggested that luminal gating of the RyRs mediated by the luminal buffer calsequestrin can cause calcium transient alternans independently of the steepness of the release-load relationship. γ is determined by the sensitivity of RyRs opening to cytosolic Ca, Ca uptake and buffering, the Ca diffusion rate, and the spacing between CRUs (Colman et al., 2017). Song et al. (2018) indicated the roles of transverse-tubular networks in CaT alternans with a cardiac cell model consisting of a three-dimensional network of CRUs. They showed that low transverse-tubular network densities when the cells were small or the CRU coupling was strong can promote CaT alternans and triggered activity. Furthermore, using a distributed model of subcellular calcium, Alvarez-Lacalle et al. (2015) showed that alternans occurs via an order-disorder phase transition which exhibits critical slowing down and a diverging correlation length and suggested novel approaches to investigate the onset of the calcium alternans instability in the heart (a schematic diagram of “3R” theory is shown in Figure 5).

#### SDA and Arrhythmogenesis Arising From Amplified Pre-Existing Tissue Heterogeneities

Pre-existing tissue heterogeneities are usually present in the heart [as shown in the Supplementary Material 2. (B) APD_80_ map, (C) Ca amplitude map]. Just as repolarization, regional differences in the spatial distribution of the APD restitution and [Ca^2+^]_i_ handling characteristics are observed across the transmural ventricular walls (from epicardium to endocardium) and from apex to base (Laurita et al., 2003), as are the amplitude and phase of alternans at a given heart rate. In their study, Gizzi et al. (2013) suggested that cardiac alternans has fully 3-dimensional dynamics and the endocardium and epicardium can show significantly different dynamics when using optical mapping to record electrical activations from the endocardium and epicardium simultaneously. Cardiac tissue usually shows electrotonic interaction due to the presence of gap junctions between the cardiomyocytes. However, when there is intercellular uncoupling under pathological conditions, the adjacent regions of the cells can more easily present their potential tissue heterogeneities. It is clear that these heterogeneities may be further amplified, which results in an increase in spatial dispersion and is a potential substrate for arrhythmias. The regional heterogeneities in [Ca^2+^]_i_ handling cause AP heterogeneities through NCX activation and eventually overwhelm the electrical coupling of the tissue. Recently, Phadumdeo and Weinberg (2020) suggested that high heart rate variability may be anti-arrhythmic because of the mitigated influence from [Ca^2+^]_i_ heterogeneity and desynchronization of the APD from [Ca^2+^]_i_ instabilities. The most convincing evidence is that cellular electrical coupling may be reduced due to fibrosis, structural barriers, or due to reduced expression and remodeling of cardiac gap junction proteins (Hsieh et al., 2016), and that pre-existing heterogeneity (such as spatial gradients in APD and [Ca^2+^]_i_) may be amplified, leading to SDA (Tse et al., 2016a).

#### SDA and Arrhythmogenesis Arising From the Autonomic Nervous System

Although alternans can be easily induced by high-frequency stimulation in isolated heart experiments lacking sympathetic and parasympathetic drive, the autonomic nervous system plays a significant regulatory role in the intact heart. Under normal conditions, the effects of sympathetic and parasympathetic nerve stimulation of the heart are mutually balanced, but with sympathetic overactivity (such as in HF, hypertension, or myocardial infarction), excessive catecholamines binds to the receptors which then change the configuration of membrane ion channels through an enzymatic reaction and result in myocardial excitation and an increased heart rate. The parasympathetic and sympathetic efferent nerves reach the heart via the vagus nerve and via the L and R stellar ganglions, respectively. This gives the experimental opportunity to selectively denervate the heart. At present, simulation and experimental studies have shown that sympathetic activity can promote alternans. In early experimental studies, Schwartz and Malliani (1975) found a fright response caused visible TWA in a child with hereditary LQTS, and subsequently evoked TWA in anesthetized cats through left stellate ganglion stimulation. Winter et al. (2018) suggested that sympathetic nerve stimulation could suppress CaT and AP alternans in the intact guinea pig heart due to accelerated [Ca^2+^]_i_ handling and increased *I*_Ks._ Similarly Tomek et al. (2017) used a detailed computational model of the canine ventricular cardiomyocyte to study the determinants of alternans in the border zone and their regulation by β-adrenergic receptor stimulation (the border zone of the viable myocardium adjacent to an infarct undergoes extensive autonomic and electrical remodeling and is prone to repolarization alternans-induced cardiac arrhythmias). They suggested that the border zone may promote SDA formation in an infarcted heart and that β-adrenergic receptor stimulation abolished alternans. Furthermore, β-blockers are known to reduce mortality and prevent SCD in patients with various cardiac diseases such as ischemic and non-ischemic cardiomyopathy. Rashba et al. (2002) found that the β-blocker esmolol reduced TWA in patients with coronary artery disease, left ventricular dysfunction, and inducible sustained ventricular tachycardia. This is consistent with an inhibitory role for β-blockade in the regulation of TWA, particularly as evidence mounts for a mechanistic link between TWA and risk of SCD in a variety of patient populations.

The concept that neural activity can exert a potential effect on arrhythmogenesis was first emphasized in 1976 by Lown and Verrier where they studied how electrical stability changes in the presence of acute myocardial ischemia. They used an animal model where there was 10 min of coronary artery occlusion followed by a sudden release, such that a consistent time process is shown in the emergence of arrhythmias and VF (Lown and Verrier, 1976) and has continued to be affirmed in contemporary literature. To successfully intervene with the role of the autonomic nervous system in arrhythmogenesis, the key point is to transform the basic mechanisms of neurol control of the cardiac rate, rhythm, and of the electrophysiological properties of myocardial and conductive tissue known from experimental research into clinical application. The possible mechanisms by which the autonomic nervous system influences arrhythmogenicity are as follows. Firstly, through autonomic-mediated accentuation of the spatial dispersion of repolarization, where the increases in heterogeneity contribute to the ECG phenotype (TWA) and arrhythmogenicity of channelopathies such as the BrS, LQTS, and that autonomic effects play a prominent role in revealing these syndromes and inducing lethal events (Zipes, 1991; Miyazaki et al., 1996). Secondly, abnormal autonomic nerve activity is a strong prognostic indicator for ventricular arrhythmias as sympathetic stimulation increases the maximum slope of restitution and electrical alternans but reduces ERP and VF threshold, whereas vagus nerve stimulation has the opposite effects (Xiong et al., 2018). With regards to the effect of autonomic interventions on atrial restitution properties, the maximal slope of the APD restitution curve does not fully explain the changes in atrial fibrillation (AF) inducibility and duration caused by autonomic interventions; spatial dispersion of APD restitution kinetics may instead be an important determinant for AF susceptibility (Fuller et al., 2016).

## Discussion

**FIGURE 6:**
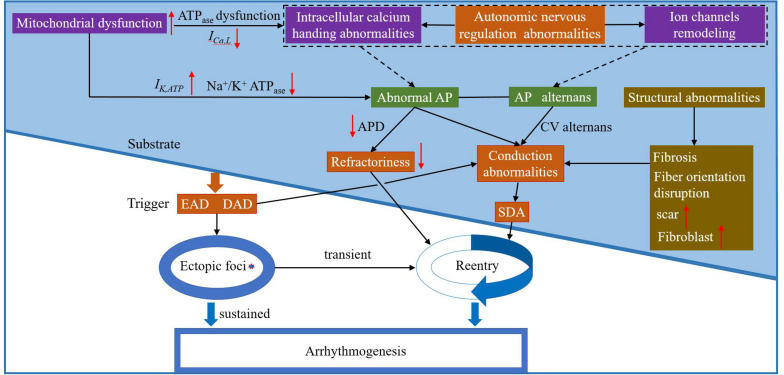
The schematic overview of arrhythmia mechanisms. Purple box: possible electrophysiological mechanism(s) of arrhythmia at the subcellular level; green box: possible electrophysiological mechanism(s) of arrhythmia at the cell level; orange box: possible electrophysiological mechanism(s) of arrhythmia at the tissue level; and brown box: possible mechanism(s) of abnormal myocardial structure leading to arrhythmia. EAD: early afterdepolarization; DAD: delayed atferdepolarization; AP: action potential; CV: conduction velocity; and SDA: spatially discordant alternans.

Basic and clinical studies investigating TWA have greatly improved our understanding of this dynamical phenomenon and its relationship to lethal arrhythmias and SCD. Figure 6 shows the schematic overview of arrhythmia mechanisms. Although alternans is only a factor that determines the eventual occurrence of arrhythmias, alternans itself is a sign of cardiac electrical instability. Understanding the mechanisms of these instabilities is vital for developing effective therapeutic strategies. These facts, combined with the close relationship between cardiac alternans, heterogeneities of repolarization, and [Ca^2+^]_i_-V_m_ coupling provide the basis for a general hypothesis about the role of TWA-mediated arrhythmogenesis in various pathophysiological conditions. Although TWA mechanisms have been partially elucidated, a clear origin of cardiac alternans (period-doubling bifurcation caused by the non-linear dependence of the APD on the previous DI, unstable calcium cycling, conduction, or tissue heterogeneity) and the spontaneous transition from spatially concordant or discordant alternans to arrhythmias remain ambiguous. Much like in HF, there is extensive ion current remodeling. HF increases the susceptibility to cardiac alternans, which is caused by HF-induced impairment in [Ca^2+^]_i_ cycling (Wilson et al., 2009). In CPVT, mutations in the RyRs lead to diastolic calcium leak and generation of discordant CaT alternans (Yang et al., 2016). LQTS are characterized by APD prolongation and increased the gradients of the APD restitution curve leading to the production of APD alternans (Gadage, 2018). In myocardial ischemia and sodium channel blockade, CV restitution may be more important in the generation of SDA (Qu et al., 2004). Therefore, more advanced experimental techniques and equipment are needed to study the mechanisms associated with TWA-mediated arrhythmogenesis and provide a theoretical basis for subsequent clinical treatment.

Currently potential mechanisms of alternans at the cellular level and SDA and their possible arrhythmogenic consequences at the tissue level have been reviewed. At the cellular level, many hypotheses have been proposed to explain the genesis of cardiac alternans: (1) Steep APD restitution curve is likely due to the actions of one or more ionic currents. Therefore, the maximum slope of the APD restitution curve can be flattened by changing the kinetics of one or more membrane currents, and this has been proved to prevent the development of cellular alternans (Qu et al., 2000); (2) However, the slope of APD restitution may fail to predict alternans when [Ca^2+^]_i_ cycling has a strong influence on AP or the presence of cardiac memory. As subcellular disruptions of [Ca^2+^]_i_ homeostasis can itself lead to alternans and the APD restitution curve becomes less steep than it was initially in the presence of cardiac memory; and (3) Furthermore, MEF indicates that alternating pulsus can contribute to electrical alternans. In the heart, numerous simulation and experimental studies have also highlighted SDA formation mechanisms including dynamic factors (CV restitution, steep APD restitution curve, or [Ca^2+^]_i_ cycling instability), pre-existing tissue heterogeneities, and regulation of the autonomic nervous system. SDA provides a substrate for unidirectional block and reentry and the manifestation of SDA is associated with increased spatial dispersion of refractoriness, wavefront fractionation, and the onset of reentrant arrhythmias.

Up to date there are currently two techniques for evaluating TWA – the MMA method and the spectral method. The spectral method can be applied for TWA detection during exercise tests, and the MMA method can be applied always for evaluation of the TWA during both exercise and Holter monitoring during daily activities. But a large number of SCD occur unrelated to exercise, and the risk of arrhythmia may be also increased by other factors like mental stress (Hekkanen et al., 2020). Thus, the MMA-TWA assessment during daily activities may be considered as a superior option. A meta-analysis published by Quan et al. (2014) on MMA-TWA including patients with both non-ischaemic and ischaemic cardiomyopathies suggested that positive MMA-TWA is associated with a 4.75-fold higher risk of cardiac mortality and 7.5-fold higher risk of SCD. However, AHA/ACC/HRS recommendations on non-invasive techniques in risk stratification underline that although the majority of the data supports the role of TWA in risk stratification, further prospective randomized trials are needed to prove the efficacy of TWA-guided antiarrhythmic therapy (Goldberger et al., 2008).

Based on TWA electrophysiological mechanisms, there are two ways to achieve upstream antiarrhythmic effects. The first way is by suppressing cardiac alternans, which would depend on the ability to detect electrical alternans regardless of where it originates from the heart and then takes the form of adaptive pacing protocols. These protocols could be incorporated into an implantable device thereby inhibiting cardiac alternans and potentially suppressing the development of a proarrhythmic substrate, such as a premature beat. This has good clinical applicability as many implantable devices (e.g., ICDs) have endocardial electrodes. Several studies have shown a close correlation between simultaneous measurements of cardiac alternans from the body surface ECGs and intracardiac EGMs, indicating that these methods can measure the same phenomena (Paz et al., 2006; Weiss et al., 2011). Compared with body surface ECGs, intracardiac EGMs have significantly larger alternans magnitude and may provide a more robust assessment of the relation between surges in cardiac alternans and short-term arrhythmia susceptibility. This has been tested by developing a novel intracardiac electrode configuration to detect cardiac alternans in a highly reproducible manner in an acute ischemia animal model (Weiss et al., 2011). The second potential way has been under extensive research over the past decade with a focus on stimulating the parasympathetic nervous system to restore autonomic imbalances. Several studies have shown that vagus nerve stimulation reduces the maximum slope of the restitution curve and the amplitude of electrical alternans, increases the VF threshold (Xiong et al., 2018), and reduces the levels of TWA in patients with drug-refractory partial-onset seizures (Schomer et al., 2014). In clinical trials, TWA has been widely used as a predictor of malignant arrhythmia and SCD, and it has been suggested that suppression of cardiac alternans can become an anti-arrhthmic therapeutic target, but there is currently insufficient evidence to guide treatment. Based on the clinical value of TWA, we believe TWA-analysis can achieve early prevention (such as detecting TWA through the use of wearable devices) and individualized treatment of arrhythmia.

Alternatively, use of antiarrhythmic drugs may provide another helpful clinical therapy for TWA treatment. Whatever the specific mechanisms for these arrhythmogenic alternans is, the ultimate common pathway for arrhythmogensis includes wave-break, conduction block, and the occurrence and maintenance of re-entrant arrhythmias (Liu et al., 2018; Colman, 2020). Numerous studies using animal models have shown that the anti-arrhythmic effects of many drugs are mediated to some extent by their effects on cardiac dynamics. These include traditional agents such as β-blockers (Rashba et al., 2002) as well as novel drugs such as late sodium current blockers (Fukaya et al., 2019), ryanodine receptor stabilizers (Lehnart et al., 2006), anti-fibrotic agents (Bathgate et al., 2008), and gap junction openers (Hsieh et al., 2016). In contrast, gap junction inhibitors can also exert antiarrhythmic effects by prolonging the ERPs (Tse et al., 2016b). Their effects on [Ca^2+^]_i_ cycling dynamics are complex, depending on the nature of Ca^2+^ → voltage coupling. The differences in CaT between adjacent cells are magnified in negative coupling but decrease with positive coupling (Sato et al., 2006). Activation of SACs exerts opposing effects on alternans, inhibiting SCA while exacerbating SDA. Therefore, SACs inhibitors can exert antiarrhythmic effects by suppressing SDA (Galice et al., 2016). However, the antiarrhythmic effects of some of the drugs described above illustrate the difficulty of predicting the overall electrophysiological effects of them. Therefore, more targeted novel drug development is necessary. Furthermore, based on the CiPA (Comprehensive *in vitro* Proarrhythmia Assay), the risk assessment of antiarrhythmic drugs using a mathematical model of the cardiac action potential is further regulated. Although CiPA initiatives incorporate cell action potentials as a component into the simulation evaluation, attempts to use mathematical simulations in drug safety evaluations are expanding, even using biophysical 3-dimensional models of the heart and torso to simulate the human ECG under drug action. Future efforts will require the combination of simulations with experimental studies so that the complex spatiotemporal properties of cardiac dynamics can be considered in different tissue-level models and provide new ideas for individualized treatment.

## Author Contributions

All authors participated in the conception and writing of the manuscript. KZ reviewed and suggested modifications to the content.

## Conflict of Interest

The authors declare that the research was conducted in the absence of any commercial or financial relationships that could be construed as a potential conflict of interest.

## Abbreviations

TWA: T-wave alternans
ECG: electrocardiogram
SCD: sudden cardiac death
APD: action potential duration
CaT: calcium transient
SCA/SDA: spatially concordant/discordant alternans
MTWA: microvolt TWA
LQTS: long QT syndromes
DI: diastolic interval
SR: sarcoplasmic reticulum
[Ca^2+^]_i_: intracellular Ca
BCL: basic cycle length
AP: action potential
VF: ventricular fibrillation
*I*_Na,L_: late sodium current
*I*_Na_: inward Na+ current
*I*_Ca,L_: L-type Ca^2+^ current
*I*_K1_: inward rectifier current
*I*_Kr_: rapid delayed rectifier potassium current
*I*_Ks_: slow delayed rectifier potassium current
*I*_to_: outward transient potassium current
BrS: Brugada syndrome
CV: conduction velocity
ERP: effective refractory period
RyR_s_: ryanodine receptors
NCX: Na-Ca exchanger
SERCA: SR-Ca-ATPase
*I*_NCX_: sodium-calcium exchanger current
CiPA: Comprehensive *in vitro* Proarrhythmia Assay
CPVT: catecholaminergic polymorphic ventricular tachycardia
EGMs: electrograms
AF: atrial fibrillation
MMA: modified moving average
SACs: stretch-activated channels
MEF: mechano-electric feedback
ICDs: implantable cardiac defibrillators
CRUs: Ca release units
HF: heart failure
[Ca^2+^]_SR_: intra-SR free [Ca^2+^].
