# Supplementary material for: Electrophysiological Mechanisms Underlying T-Wave Alternans and Their Role in Arrhythmogenesis
Source: Front Physiol. 2021 Mar 4;12:614946. doi: 10.3389/fphys.2021.614946 (PMC7969788; doi:10.3389/fphys.2021.614946)
Supplement: Supplementary file 1 [file Data_Sheet_1.DOCX]

**Supplementary materials**

**Experimental methods of patch clamp and optical mapping**

**1. Whole-cell patch clamp in guinea-pig ventricular myocytes**

**1.1 Myocyte isolation**

Ventricular myocytes were isolated from male guinea pig (250-350g, Laboratory Animal Center, Southwest Medical University). The animals were housed (3 per cage) under conditions of controlled temperature (23-25°C) and humidity (55-65%) with a 12-h light/dark cycle. The animals had ad libitum access to water and food. Guinea pig hearts were isolated by thoracotomy after intraperitoneal injection of heparin (3125 UI/kg) and sodium pentobarbital (50 mg/kg). The depth of the anesthesia was evaluated by foot pinch or checking corneal reflexes to assure a deep anesthesia and that the animal does not experience any pain. Hearts were excised, mounted on a Langendorff apparatus (Harvard Apparatus, Holliston, MA) and retrogradely perfused via the aorta. After an initial 2-3 min perfusion with oxygenated (100% O_2_) Tyrode solution (37℃) containing (mmol/L): 140 NaCl, 5 KCl, 1 MgCl_2_, 10 D-Glucose, 5 HEPES, 1.8 CaCl_2_ (pH adjust to 7.35 with NaOH) in constant flow rate (8ml/min). Thereafter, Ca^2+^-free Tyrode solution was perfused for 8–10min, followed by a solution containing 0.02% collagenase (Type II, Worthington Biochemical, Lakewood, NJ, USA) and 0.15% bovine serum albumin. When the heart became softened the ventricular was dissected from the heart and minced in a oxygenated high-K^+^ storage medium containing ( mmol/L): 10 KCl, 10 KH_2_PO_4_, 120 K-glutamate, 10 taurine, 1.8 MgSO_4_, 10 HEPES, 20 glucose, 10 mannitol, 0.5 EGTA, as well as 0.2% albumin (PH adjust to 7.3 with KOH) at room temperature, and then used within 10h. All chemicals and reagents were from Sigma-Aldrich (St. Louis, MO, USA), unless otherwise stated.

**1.2 Patch clamp and data analysis**

A whole-cell patch clamp technique was employed to record action potentials (AP_s_) in current-clamp mode with an Axopatch 700B amplifier and the Axon Digidata 1440A interface (Axon Instruments, Foster City, CA). AP recordings were lowpass filtered at 5 kHz and digitized at 10 kHz. All patch clamp experiments were performed under identical conditions (e.g. similar patch duration for each protocol) at 37℃. Patch clamp pipettes (2.5-5 MΩ filled with internal solution) were pulled from borosilicate glass capillaries (WPI, Sarasota, FL, USA) with a horizontal puller P-97 (Sutter Instruments, Novato, CA, USA). For all current-clamp experiments, pipettes were filled with internal solution containing ( mmol/L): 20 KCl, 110 K-aspartate, 10 HEPES, 5 EGTA and 5 Na_2_-phosphocreatine, 0.1 NaGTP, 5 MgATP (pH adjusted to 7.2 with KOH). The chamber was perfused with Tyrode solution containing (mmol/L): 140 NaCl, 5 KCl, 1 MgCl_2_, 10 D-Glucose, 5 HEPES, 1.8 CaCl_2_ (pH adjusted to 7.35 with NaOH). AP_s_ were evoked by 4ms stimulation pulses with a magnitude 1.5 times higher than AP activation threshold.

Standard stimulations (S1S2 protocol) involved an application of a train of 15 regular pulses at stimulus interval of 500ms, followed by a premature extra-stimulus generated at progressively reduced coupling intervals. The stimulations were started with a coupling interval of 350ms, which was then decreased with the step of -10ms, until the refractoriness was reached. Then the smallest coupling interval that diastolic interval (DI) is less than 0ms was recorded and used to calculate effective refractory period (ERP). The APD_80_ values were determined either in the premature extra-stimulus-evoked AP or in the last 5 APD_80_ averages in a train of regular pulses, and then plotted as a function of the preceding DI. The restitution curves were fitted using an exponential function. The maximum restitution slopes were determined in each experiment from the first derivative of the exponential fit. Dynamic stimulations (S1S1 protocol) were used to evoke AP alternans, starting with a stimulus interval of 350ms and gradually increasing the frequency with the step of -10ms until the onset of 2:1. APD calculations were performed using Clampfit 10.7 (Molecular Devices, USA). Non-linear curve fitting was performed using OriginPro 8.0 (Origin Lab, USA).

**2. Dual optical mapping in isolated guinea-pig hearts**

**2.1 Whole-heart preparation and dyes loading**

Male guinea pigs (250-350g) were anaesthetized with an intraperitoneal injection of heparin (3125 UI/kg) and sodium pentobarbital (50 mg/kg). The depth of the anesthesia was evaluated by foot pinch or checking corneal reflexes to assure a deep anesthesia and that the animal does not experience any pain. Hearts were rapidly excised, mounted on a Langendorff apparatus and retrogradely perfused via the aorta. The heart was perfused with modified Krebs-Henseleit solution containing (mmol/L): 119 NaCl, 25 NaHCO_3_, 4 KCl, 1.2 KH_2_PO_4_, 1 MgCl_2_, 10 D-Glucose, 1.8 CaCl_2_. The medium was continuously oxygenated with a 95% O_2_/5% CO_2_ gas mix with the flow rate of 8 ml/min at 37±0.2°C. Isolated hearts were perfused and monitored for stability for 20 mins before experimental procedures commenced. After the Langendorff-perfused hearts reached steady state, contraction artefacts were mimimized using blebbistatin (10 µM, Abcam). Dye loading was aided by pre-perfusion with pluronic F127 (20 % w/v in DMSO, Invitrogen). Voltage-sensitive dye RH237 (1µg/ml, Santa) and calcium sensitive dye Rhod2-AM (1µg/ml, Abcam) were perfused from drug port to enable simultaneous membrane potential and Ca^2+^ measurements at 37±0.2 ℃.

**2.2 Optical mapping system and data acquisition**

Two LEDs with bandpass filters (wavelength 530±20nm, LEDC-2001, MappingLab Ltd) were used for excitation. The fluorescence light was first passed through a 550nm long-pass filter, then through a dichroic mirror by cutting off 638nm. Voltage signals were recorded with a 700nm long-pass filter which passed through fluorescence light with wavelengths above 638nm. Calcium signals were recorded with a bandpass filter (585±40nm) which passed through fluorescence light with wavelengths below 638nm (OMS-PCIE-2002, MappingLab Ltd). Movies were acquired at 900 frames/second, with 128-by-128 pixels resolution (1.5625µm-square per pixel). The cameras of the optical mapping system and the LED lamp were driven by OMapRecord 4.0 software (MappingLab Ltd.) simultaneously. Omapscope5.0 software (MappingLab Ltd) was used for semi-automatic processing to analyze the optical mapping data and generate isochron mapping.

Electrical Stimuli were generated by an isolated constant voltage/current stimulator (VCS3001, MappingLab Ltd., UK). They were delivered with a platinum electrode onto the epicardial apex at an amplitude 1.5 × the diastolic voltage threshold and a 2ms pulse width. To induce cardiac alternans stimulation frequency was gradually increased by an incremental fast pacing protocol (a train of 50 pulses) applied at progressively reduced stimulation intervals. In successive stimulations, the S1-S1 interval was reduced from 350ms (the longest pacing interval producing no ventricular escaped beats) down to the values (about 150ms) that induced 2:1 response or even VF under basal conditions. For each pacing rate, a consecutive movie of 7-10 seconds was recorded. Pacing protocol and movie recording was automatized to ensure the shortest duration of the experiment. In case VF was induced, pacing protocol was finalized. Optical action potential duration (APD_s_) was calculated as 80% of repolarization. Calcium transient (CaT) magnitude was measured as the peak-to-peak amplitude of CaT. In this experiment, stable recordings for intracytoplasm Ca^2+^ and voltage (V_m_) signals could be obtained for >1.5 hours in preliminary experiments assessing system stability.


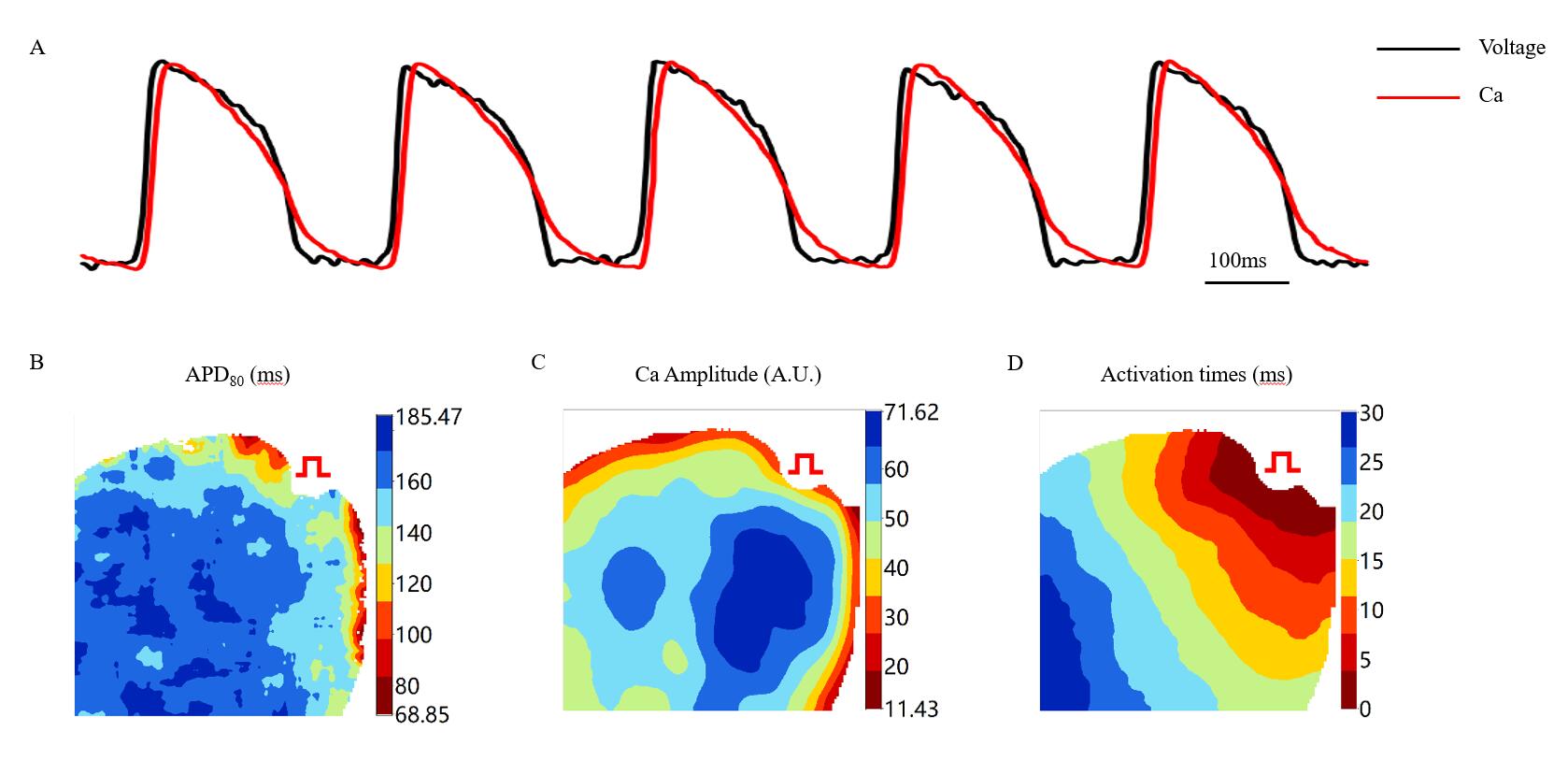


Figure S1 A. a schematic diagram of voltage/Ca signal of single pixel, B. APD_80_ map, C. Ca amplitude map and D. activation times map (measured by optical mapping in isolated guinea pig hearts; Stimulus :2-fold threshold, apex.)


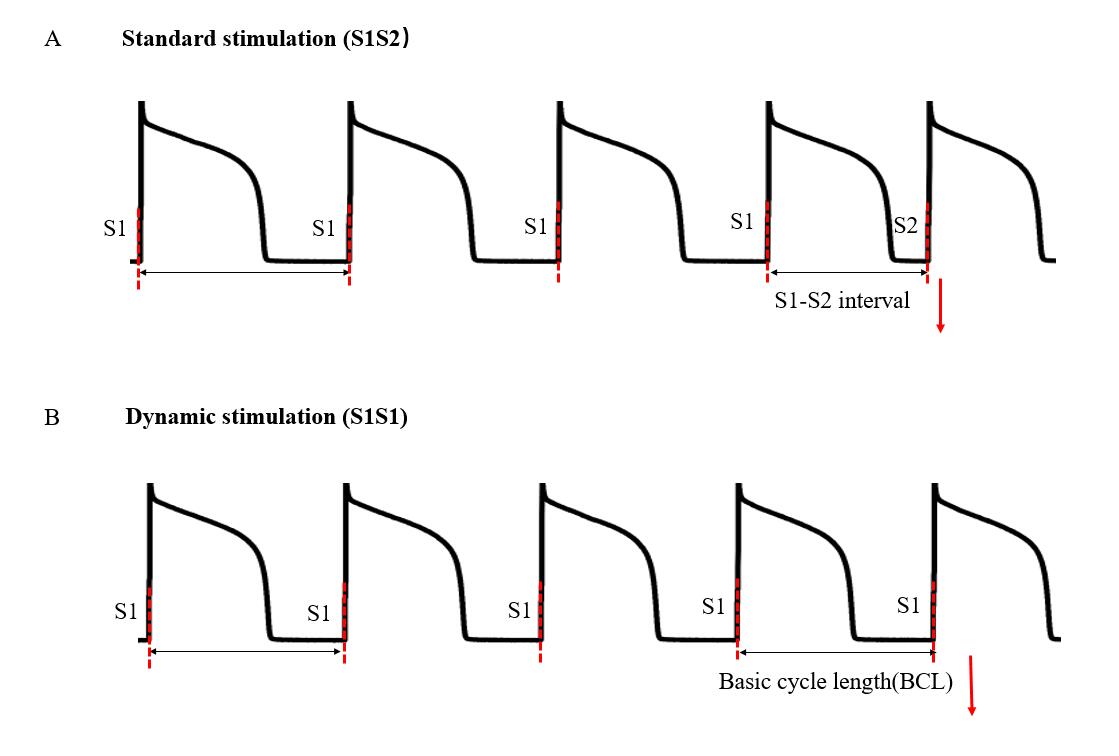


Figure S2 Stimulation protocols used to measure APD restitution curve. S1S2: an extrastimulus application at different coupling intervals after a series of regular pacing pulses; S1S1: steady pacing at gradually increasing rates.
